# Comparison of photosynthetic activity and heat tolerance between near isogenic lines of wheat with different photosynthetic rates

**DOI:** 10.1371/journal.pone.0255896

**Published:** 2021-12-13

**Authors:** Chongyang Li, Mingyang Ma, Tianpeng Zhang, Pengwen Feng, Xiao Chen, Yang Liu, Marian Brestic, Tarek M. Galal, Hatim M. Al-Yasi, Xinghong Yang

**Affiliations:** 1 College of Life Science, State Key Laboratory of Crop Biology, Shandong Key Laboratory of Crop Biology, Shandong Agricultural University, Taian, China; 2 Department of Plant Physiology, Slovak University of Agriculture, Nitra, Slovak Republic; 3 Department of Biology, College of Sciences, Taif University, Taif, Saudi Arabia; Huazhong University of Science and Technology, CHINA

## Abstract

Wheat (*Triticum aestivum* L.) is one of the most important crops in the world, but the yield and quality of wheat are highly susceptible to heat stress, especially during the grain-filling stage. Therefore, it is crucial to select high-yield and high-temperature-resistant varieties for food cultivation. There is a positive correlation between the yield and photosynthetic rate of wheat during the entire grain-filling stage, but few studies have shown that lines with high photosynthetic rates can maintain higher thermotolerance at the same time. In this study, two pairs of wheat near isogenic lines (NILs) with different photosynthetic rates were used for all experiments. Our results indicated that under heat stress, lines with a high photosynthetic rate could maintain the activities of photosystem II (PSII) and key Calvin cycle enzymes in addition to their higher photosynthetic rates. The protein levels of D1 and HSP70 were significantly increased in the highly photosynthetic lines, which contributed to maintaining high photosynthetic rates and ensuring the stability of the Calvin cycle under heat stress. Furthermore, we found that lines with a high photosynthetic rate could maintain high antioxidant enzyme activity to scavenge reactive oxygen species (ROS) and reduce ROS accumulation better than lines with a low photosynthetic rate under high-temperature stress. These findings suggest that lines with high photosynthetic rates can maintain a higher photosynthetic rate despite heat stress and are more thermotolerant than lines with low photosynthetic rates.

## Introduction

Temperature variability is one of the most important conditions affecting agricultural production. With the rise in global temperatures and extreme weather conditions, high temperatures significantly affect the quality and quantity of crops [1, 2]. Wheat is an important crop, but as global temperatures rise, wheat production has seriously suffered, especially in the grain-filling stage, resulting in reduced yield and quality of wheat. The optimum temperature for wheat growth in the grain-filling stage is approximately 21°C [3, 4].

The effect of heat stress on wheat productivity is quite complex. Photosynthesis is the most vulnerable to high-temperature physiological processes [5–9] and any drop in photosynthesis would impact wheat growth and yield [10, 11]. Chloroplast enzyme inactivation, which is largely attributable to oxidative stress, can also decrease the photosynthesis rate in leaves. Oxidative stress may also cause lipid peroxidation, which leads to protein degradation, membrane disorder and enzyme inactivation. Photosystem II (PSII) can be affected by temperatures above 45°C [12]. The PSII repair mechanism is hampered, minimizing carbon fixation and oxygen discharge, thereby upsetting linear electron flow [3] because reactive oxygen (ROS) damage occurs in the de novo synthesis system. The D1 protein (encoded in the transcript of *PsbA*), which defends PSII against oxidative damage, is an essential part of the photosynthesis process in higher plants [13]. Huo, Wang [14] showed that during drought stress, plants overexpressing D1 protein showed much lower reductions in the photosynthetic rate (Pn), stomatal conductance (Gs), and maximal photochemical efficiency (Fv/Fm) of PSII than wild-type plants. Nonphotochemical quenching (NPQ) was negatively correlated with ROS under excess light energy conditions [15, 16], and weakening NPQ promoted ^1^O_2_ production in PSII [17]. The use of fluorescent chlorophyll will help distinguish heat-resistant or vulnerable lines for further study or as a means of resistance to higher temperatures for crop growth [18]. The improved photosynthetic performance of modern wheat varieties suggests that the long-term process of wheat selection and breeding toward high yield and optimum phenotypes has contributed to the optimization of photosynthetic processes [19].

Heat stress causes the production and accumulation of ROS, contributing to oxidation of cell membranes, protein oxidation and damage to DNA [20–27]. Therefore, detoxification by the antioxidant system is important to protect plants under heat stress [28, 29]. To cope with adverse heat stress–induced effects, plants have evolved complex defense strategies, such as osmolyte accumulation (e.g., sucrose, betaine, and trehalose) and ROS scavenging systems, which consist of enzymatic and nonenzymatic mechanisms [24–27]. Various scientists have shown that heat stress-induced harm to plants can be eased with antioxidant systems such as superoxide dismutase (SOD) and peroxidase (POD) [30, 31]. In addition, under a variety of stressors, the important roles of reduced ascorbate (AsA) and glutathione (GSH) in the AsA-GSH cycle have also been reported [24–27, 32].

There are few studies showing that lines with a high photosynthetic rate can also tolerate high temperatures [18, 33]. However, the differential genetic makeup of wheat NILs increases the difficulty of further studying the key processes controlling photosynthesis and the sites thereof. Xiaoyan 54, a winter wheat, has strong temperature resistance, while 8602 is a high-yield variety. Their offspring produced lines with high photosynthetic rate lines (154 and 23) and lines with low photosynthetic rate lines (212 and 94). Through this study, we expect to determine whether lines with high photosynthetic rate lines also have high-temperature stress resistance, which would provide a theoretical basis for screening highly photosynthetic and highly thermotolerant varieties.

## Materials and methods

### Plant material and growth conditions

The progeny F6 selected for Xiaoyan 54 and 8602, using photosynthesis (Pn) as the index of physiological range, were wheat NIL (lines 154 and 212 and lines 23 and 94). Lines 154 and 23 had high rates of photosynthesis. NILs were genetically unchanged after six generations and exhibited no differentiation. Plump seeds were produced in flower pots in the fields of the Shandong Agricultural University Experimental Station during the rising seasons of 2018 and 2019. Six 4 m^2^ rows, where rows were 25 cm and lines were 5 cm, were interspersed in each section. The diameter of the flowerpots was 30 cm, and the depth was 34 cm. The wheat was placed into the culture room and grown to the flowering and grain-filling stages. Flag leaves were used for the analyses.

High-temperature stress conditions were as follows: temperature, 42°C; light intensity, 500 μmol m^−2^ s^−1^; and relative humidity, 70%. After the high-temperature treatment, the flag leaves were frozen in liquid nitrogen for 30 min and placed in a −80°C ultralow-temperature refrigerator.

### Measurement of the photosynthetic gas exchange parameters of flag leaves

The Pn, intercellular CO_2_ (Ci), and CIRAS-3 portable photosynthetic method (PP Systems, Hitchin, USA) were calculated with intact wheat flag leaves. The relative air humidity was set to 70%, and the cuvette temperature was set to 25°C. The air flow rate was fixed to 250 μmol s^−1^ by the cuvette. The CO_2_ level at 380 μmol mol^−1^ was regulated.

### Measurements of chlorophyll fluorescence

An FMS-2 compact fluorometer (Hansatech) was used to measure chlorophyll fluorescence at room temperature (25°C). With white actinic light at an irradiance of 400 μmol.m−2s−1, the leaves were constantly illuminative. The fluorescence (Fs) steady-state value was reached in approximately 6 minutes. A second saturating pulse of 8.000 μmol m^-2^ s^-1^ was applied after the measurements were taken. The optimum degree of fluorescence was then calculated in an adapted light state (Fm). For each biological study, five replicates were used.

PSII was determined with the following: the real photochemical efficiency of a light-adapted state (Ф PSII).

ФPSII = (Fm’ − Fs)/Fm’

NPQ = (Fm − Fm’)/Fm’

qP = (Fm’ − Fs)/(Fm’ − Fo’).

### Measurement of D1 protein and HSP70

The thylakoid membrane was extracted from the flag leaves to determine the expression level of D1 protein. Leaves were homogenized with HMSN extract (0.01 M HEPES (pH-7.6), 0.4 M sucrose, 0.01 M NaCl, 0.005 M MgCl_2_, 5 mM EDTA-Na_2_, 5 mM EGTA). The homogeneous product was purified by three filtering layers and centrifuged at 4°C at 5000 g for 10 minutes. Thereafter, pigment content was determined by suspending 50 μL of thylakoid membrane in enough 95% ethanol to reach 5 mL. The absorption of solvent was measured with concentrations of 665 nm, 649 nm and 470 nm, and the pigment. We then adjusted the pigment concentrations of the samples to be consistent with one another. The remaining extracted thylakoid membrane was added to a 1/5 volume of loading buffer and boiled.

In leaves from which the HSP70 protein was extracted, the combination of 0.5 M Tris–HCl (pH 6.8), 2% (v/v) (pH), 2% (w/v) sulfate (SDS), 5% (v/v) β–mercaptoethanol, and 0.01% (w/v) bromophenol blue were used. The quantified protein was then denatured at 95°C for 3–5 minutes in a water bath and processed before testing at −20°C. Electrophoresis SDS-polyacrylamide gel electrophoresis (SDS-PAGE) was performed using a 5% stacking gel and 12% separating gel, as defined by Laemmli [34].

Western blot analysis was used to measure the expression levels of D1 and HSP70. A 0.22 μm PVDF membrane was transferred and observed with antibodies raised against D1. Proteins (15 μg per sample) were isolated with SDS-PAGE. For 2 h, the membrane was blocked with 5% nonfat dry milk and washed three times with PBS. A rabbit anti-PsbA antibody (1:3,000 dilution) was used to monitor the membrane for PBS, supplementing with 5% nonfat dried milk. The membrane was washed with PBS and incubated with goat anti-rabbit IgG hRP-conjugates in PBS at room temperature (25°C) for 1 hour, supplemented with 5% nonfat dried milk after overnight incubation at 4°C. The membrane was washed and incubated three times with PBS (TransGen Biotech) and with a Western blot package.

### RNA isolation and qRT-PCR

As defined by the TRI reagent protocol (TIANGEN), total RNA was extracted from leaves. For all samples, the complete RNA (l μg) for RT-PCR was translated by the manufacturer according to directions for the cDNA for RT-PCR with TransScript one-stroke gDNA Elimination and cDNA Synthesis SuperMix kit (TransGen Biotech).

Quantitative real-time PCR (qRT-PCR), used as an internal control, was performed to evaluate expression levels using primers developed and synthesized by Sangon Biotech (Shanghai) Co., Ltd. through NCBI. The PCR primers were engineered to avoid retained areas and give 150–200 bp products. S1 Table details the sequences.

The qRT-PCR was performed using TransStart TipTop Green qPCR SuperMix (Transgen, China). Multiple primers were mixed and amplified to test whether they were suitable. The specificity of the primers was verified using a melting curve. A single peak was considered to be specific amplification, and the annealing temperature and amount of primers were adjusted accordingly. Using actin as the internal reference, we adjusted the concentration of each template so that the difference between the values of the internal parameters of the threshold cycle (C_t_) was less than 2. Each gene was amplified simultaneously with the internal reference. The C_t_ value was read by default, and each sample was retested three times. The relative abundance of 2^−ΔΔCT^ and the internal reference was calculated to determine the level of gene expression. The instrument used was the CFX96 Real-Time PCR System (Bio-Rad).

### Determination of superoxide radicals and hydrogen peroxide

The development rate of O_2_·^−^ was estimated by Elstner [35] in the presence of O_2_·^−^ by regulating nitrite formation by hydroxylamine. At 530 nm, the absorption was read.

Patterson, MacRae [36] previously studied H_2_O_2_. Cold acetone (4°C) was homogenized in 3 mL of leaf tissue (0.5 g) and centrifuged at 2000 g for 10 minutes at 4°C. Then, the supernatant (1 mL) was applied with 0.1 mL of titanium reagent (20% Ti(SO_4_)_2_). In conjunction with Hi, Ti–H_2_O_2_, 0.2 mL of 17 M ammonia solution was then applied to the complex. The precipitate was washed five times with acetone, and 5 mL of 2 M H_2_SO_4_ was dissolved. At 415 nm, the solvent absorbance was calculated. H_2_O_2_ was measured under the same treatment using a regular H_2_O_2_ curve.

### Determination of antioxidant enzyme activity

Enzyme extracts were prepared as described by Osipova, Permyakov [37], except for APX, for which 1 mM ascorbate was included in the homogenization buffer. Enzyme activity assays were performed with a UV-visible spectrophotometer (UV-2550, Shimadzu, Kyoto, Japan).

An SOD activity assay was performed according to the protocol of Dhindsa, Plumb-Dhindsa [38]. One unit of SOD activity was defined as the amount of the enzyme required to cause 50% inhibition of the reduction of NBT, which was estimated by monitoring the absorbance at 560 nm.

The activity of CAT was measured by monitoring the consumption of H_2_O_2_ (extinction coefficient 39.4 mM^-1^ cm^-1^) at 240 nm for 3 min according to the method of Teranishi, Tanaka [39].

The activity of APX was measured by the change in absorbance at 470 nm due to guaiacol oxidation according to the method of Nakano and Asada [40], and the activity of POD was measured by monitoring the rate of H_2_O_2_-dependent oxidation of ascorbate at 290 nm for 3 min according to the methods of Dhindsa, Plumb-Dhindsa [38] and Zhang and Qu [41].

### Enzyme activity and composition determination of the AsA-Glu cycle

A package from Suzhou Comin Biotechnology Co., Ltd., was used to determine the enzyme activities of monodehydroascorbate reductase (DHAR) (EC 1.8.5.1) and glutathione reductase (GR) (EC 1.6.4.2). A package was produced from Suzhou Comin Biotechnology Co., Ltd. to measure the usage of GSH, glutathiol (GSSG), ascorbic acid (ASA), and dehydroascorbic acid (DHA).

### Analysis of key enzymatic activities of the Calvin cycle

According to Sawada, Sato [42], Rubisco operation was measured with certain adjustments. The enzyme was suspended in amplification medium to assess the overall function of Rubisco. The operation as defined by Holaday, Martindale [43] was derived from sFBPase and cFBPase. As mentioned by Degl’Innocenti, GAPDH was extracted and tested [44]. The operation, defined in Haake, Zrenner [45] with the use of NADPH oxidation as a proxy, was isolated and analyzed.

### Statistical analysis

Six steps were performed with three biological replicates for all tests. Excel 2016 (Microsoft, Redmond, WA) was used for data preprocessing, and SigmaPlot 10.0 (Systat App, Erkrath, Germany) was used to produce these estimates. SPSS (SPSS, Chicago, USA) was used for statistical analysis. To check statistical significance, one-way ANOVA and post hoc Tukey’s HSD test at P = 0.05 were applied.

## Results

### Photosynthetic characteristics and physiological parameters of NILs under normal conditions

**Fig 1:**
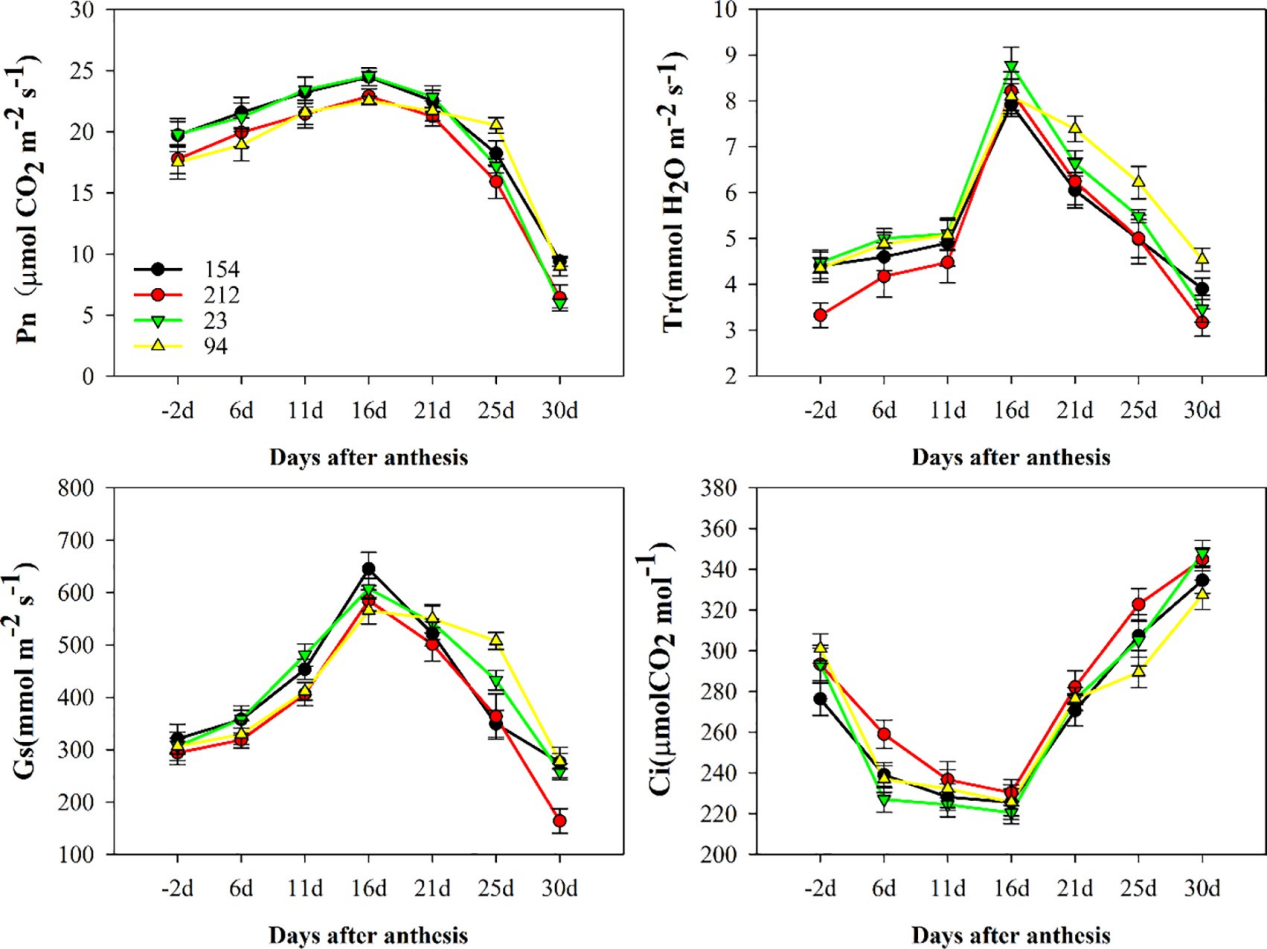
The gas exchange parameters of flag leaves in the two pairs of NILs: (A) net photosynthetic rate, Pn; (B) transpiration rate, Tr; (C) stomatal conductance, Gs; and (D) intercellular CO_2_ concentration, Ci. Lines 154 and 23 possess high photosynthetic rates; Lines 212 and 94 possess low photosynthetic rates. The values are the mean ± SE from three independent experiments.

Under normal conditions, between two pairs of near isogenic wheat lines with different photosynthetic rates (154, 212, 23, and 94), it was found that the lines with high photosynthetic rates (154 and 23) maintained their photosynthetic rate (Pn) better than the lines with low photosynthetic rates (212 and 94) until 16 days after anthesis (DAA) during the whole flowering and grain-filling stages (Fig 1A). However, at 16 DAA, there was a significant decrease in Pn in all genotypes. Stomatal conductance (Gs) and transpiration rate (Tr) showed the same trends as Pn (Fig 1A–1C), although there were evident increases in intercellular CO_2_ concentration (Ci) in all plants (Fig 1D). Furthermore, the high photosynthetic rate lines were able to maintain higher Calvin cycle key enzyme activity (such as Rubisco, GAPDH, sFBPase) in flag leaves than the low photosynthetic rate lines, which contributed to the higher Pn in the high photosynthetic rate lines (S1 Fig).

### Photosynthetic parameters of NILs under high-temperature stress

**Fig 2:**
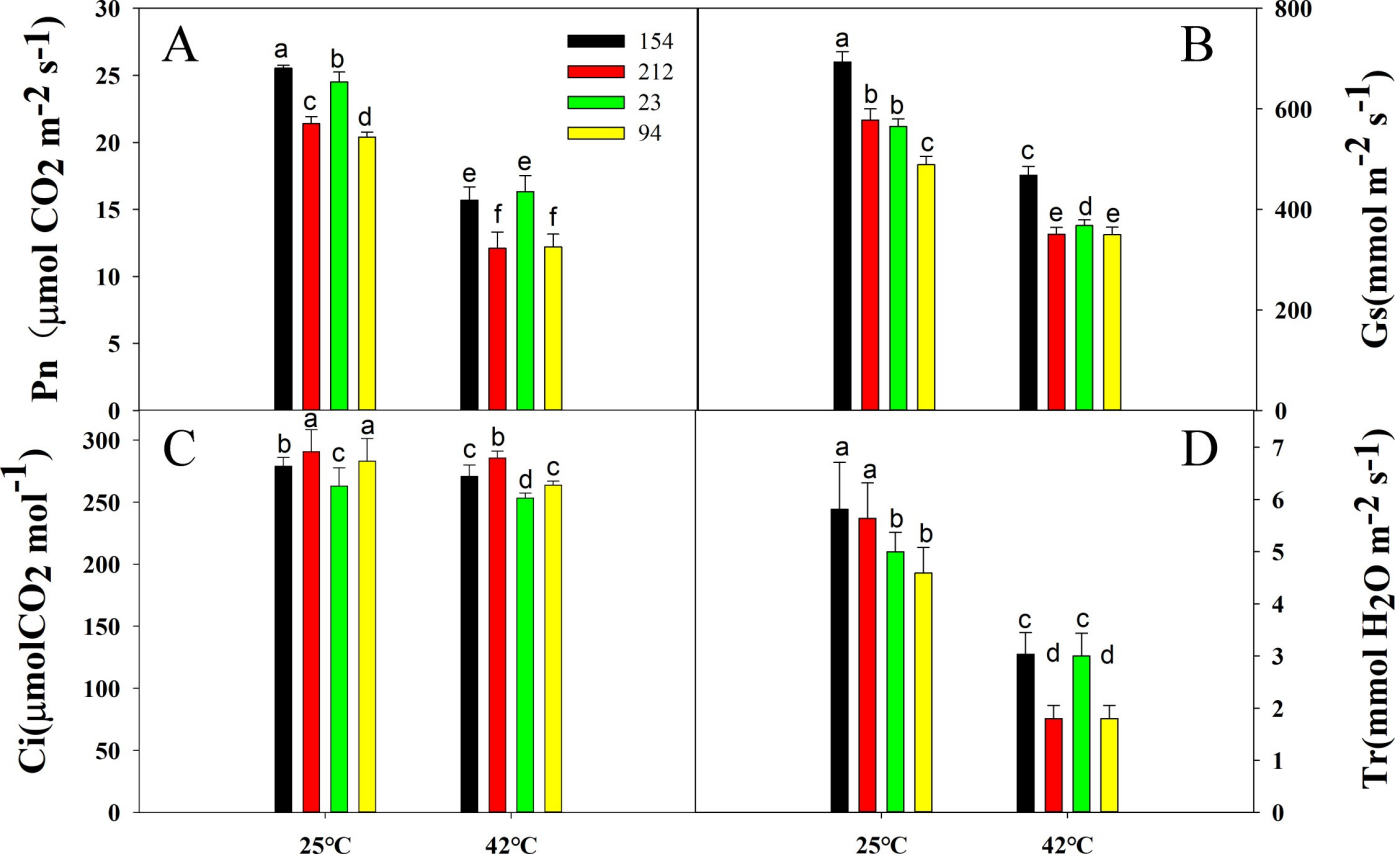
Effects of heat stress (treatment at 42°C for 4 h) on (A) Pn, (B) Tr, (C) Gs, and (D) Ci in flag leaves of NILs after anthesis. Lines 154 and 23 possess high photosynthetic rates; Lines 212 and 94 possess low photosynthetic rates. The values are the mean ± SE from three independent experiments. Different letters indicate significant differences at P = 0.05.

To evaluate the differences in photosynthesis between the two pairs of wheat NILs in response to heat stress, we selected the 11^th^ day of the grain-filling period for high-temperature treatment, which was based on the photosynthetic parameters and physiological data of all wheat plants during the grain-filling stage. We determined the CO_2_ exchange parameters and found that although Pn declined in all genotypes under high-temperature stress, the lines with high photosynthetic rates still maintained an overall higher photosynthetic rate (Fig 2A). Similar changes were found in Gs and Tr (Fig 2B and 2D). Interestingly, under heat stress, Ci also decreased in both NILs, but the Ci of lines 154 and 23 was still lower than that of lines 212 and 94 (Fig 2C).

**Fig 3:**
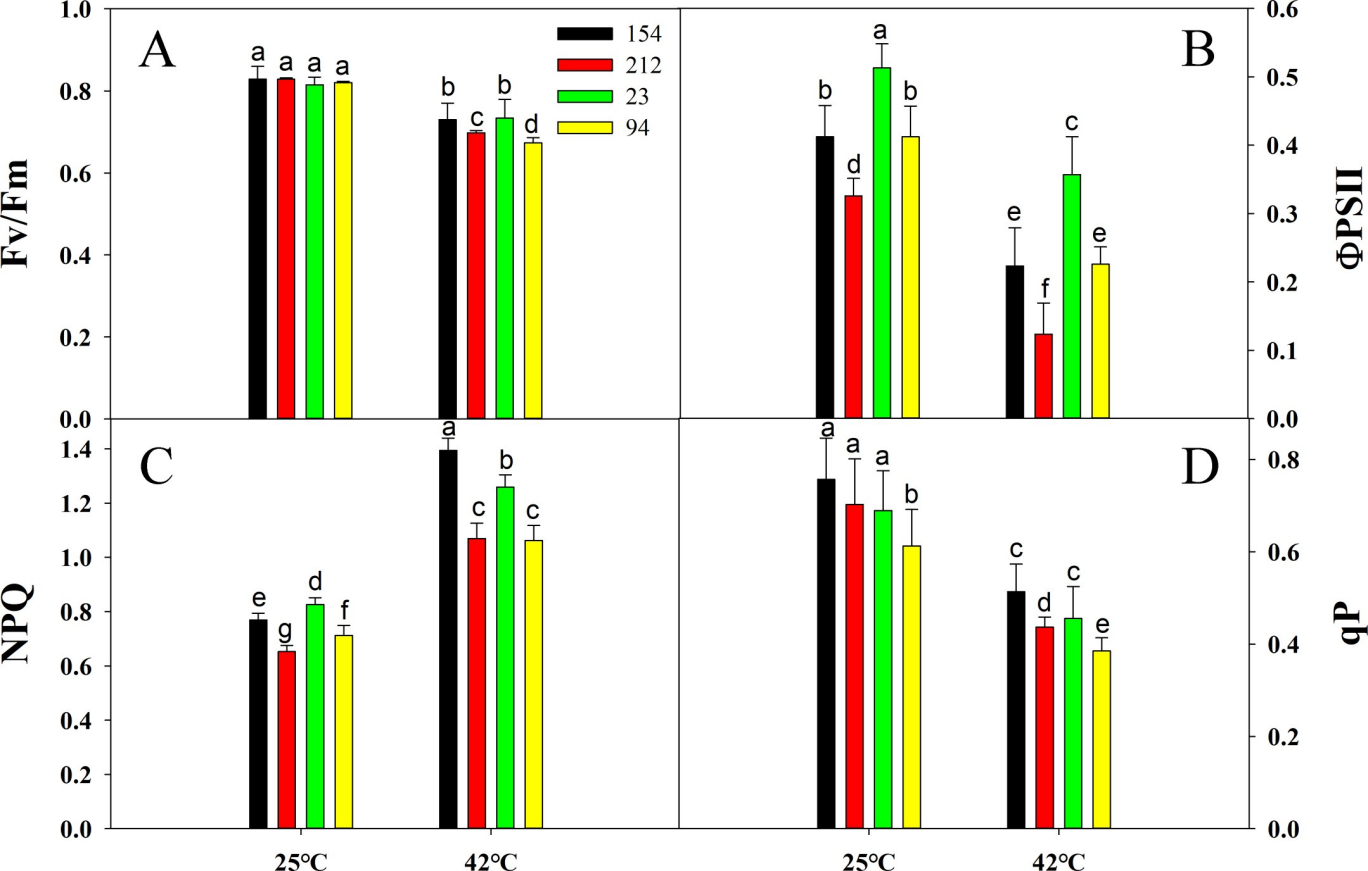
Effects of heat stress (treatment at 42°C for 4 h) on (A) Fv/Fm, (B) actual photochemical efficiency (ՓPSII), (C) nonphotochemical quenching (NPQ), and (D) qP in leaves of NILs. Lines 154 and 23 possess high photosynthetic rates; Lines 212 and 94 possess low photosynthetic rates. The values are the mean ± SE from three independent experiments. Different letters indicate significant differences at P = 0.05.

Furthermore, to investigate the effect of high temperatures on PSII photoinhibition, Fv/Fm, ՓPSII, NPQ, and qP were determined. Under normal conditions, Fv/Fm showed non-significant alterations between the four genotypes (Fig 3A). However, under heat stress, the values of Fv/Fm, ՓPSII, and qP diminished in all types of wheat, but the high photosynthetic rate lines (154 and 23) still maintained higher values of Fv/Fm, ՓPSII, and qP than the low photosynthetic rate lines (212 and 94) (Fig 3A, 3B and 3D). NPQ plays an important role in PSII light protection and is an indicator of excess excitation energy. As shown in Fig 3C, high temperatures caused obvious increases in NPQ in all wheat plants, but the NPQ of 154 and 23 lines was still higher than that of the 212 and 94 lines.

These results demonstrate that a lower degree of photoinhibition occurs in lines with high photosynthetic rates under heat stress and that the lines with high photosynthetic rates (154 and 23) can dissipate excess energy more effectively, thereby protecting PSII and maintaining a high photosynthetic capacity.

### The activity of key enzymes in the Calvin cycle under high-temperature stress

**Fig 4:**
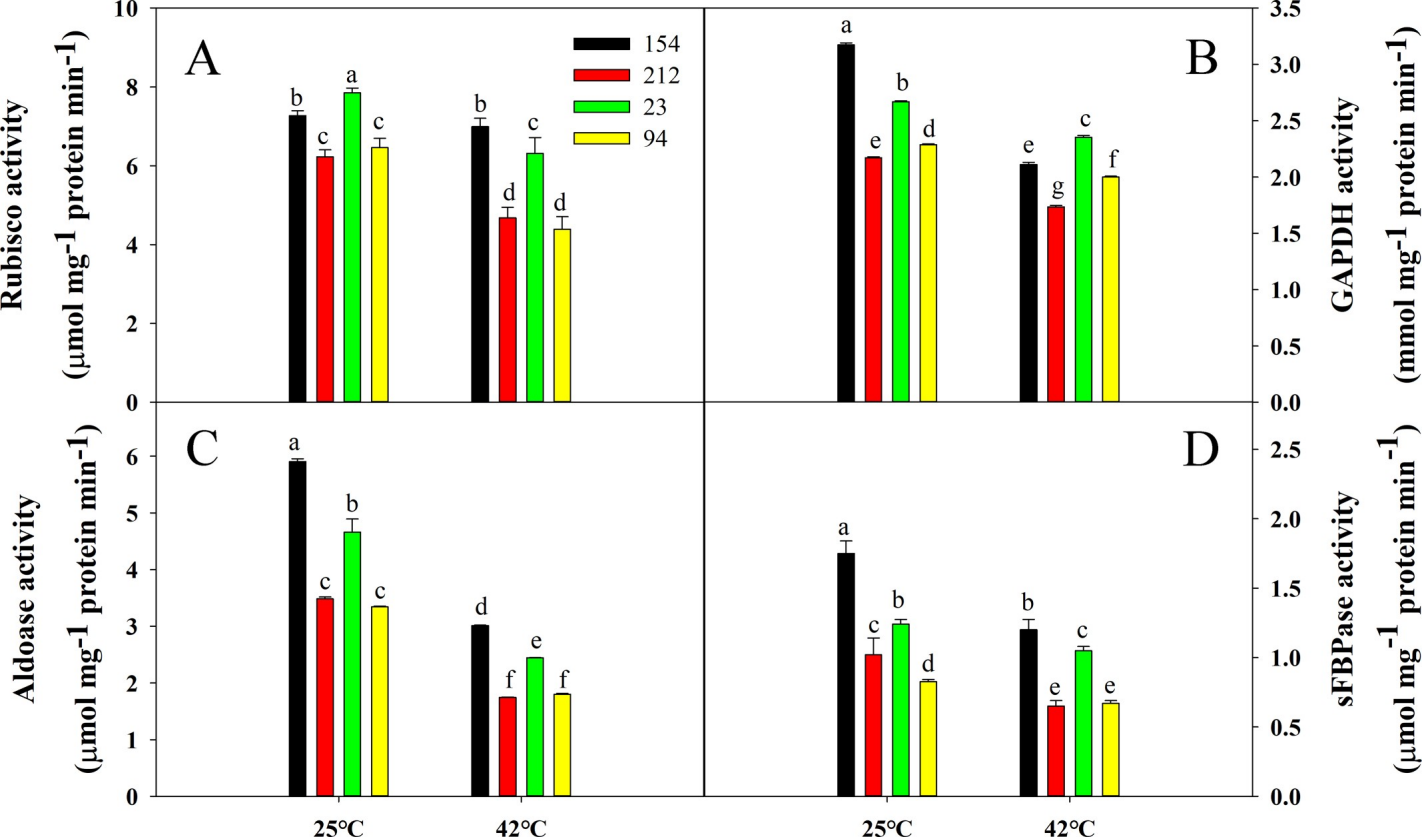
Effects of high temperature (treatment at 42°C for 4 h) on the activities of key enzymes involved in the Calvin cycle of wheat leaves: (A) Rubisco, (B) GAPDH, (C) sFBPase, and (D) aldolase. Lines 154 and 23 possess high photosynthetic rates; Lines 212 and 94 possess low photosynthetic rates. The values are the mean ± SE from three independent experiments. Different letters indicate significant differences at P = 0.05.

To further investigate why lines with a high photosynthetic rate could maintain a higher Pn under heat stress, we measured the activity of key enzymes (Rubisco, aldolase, GAPDH, sFBPase) in the Calvin cycle. Although enzymatic activity declined in all genotypes under high-temperature stress, the high photosynthetic rate lines (154 and 23) still maintained higher activity of these enzymes than the low photosynthetic rate lines (212 and 94) (Fig 4).

The 4-hour thermal stress of the wheat leaves was used for RT-PCR to evaluate the relative transcription levels of the five genes involved in the Calvin cycle: *RbcL*, *RbcS*, *FBPase*, *PGK*, and *GAPDH*, using *actin* as the reference gene. As expected, the transcription levels of these genes significantly decreased in all wheat plants under heat stress, but the transcription levels in high photosynthetic rate lines (154 and 23) were found to be significantly higher than those in low photosynthetic rate lines (212 and 94) (S2 Fig). Therefore, the expression of these genes was consistent with the trend of Calvin cycle enzymatic activity, suggesting that the high photosynthetic rate lines could maintain the higher activity of key Calvin cycle enzymes under heat stress, thereby maintaining a higher photosynthetic rate and carbon fixation rate.

### Accumulation of D1 protein and HSP70 in NILs under high-temperature stress

**Fig 5:**
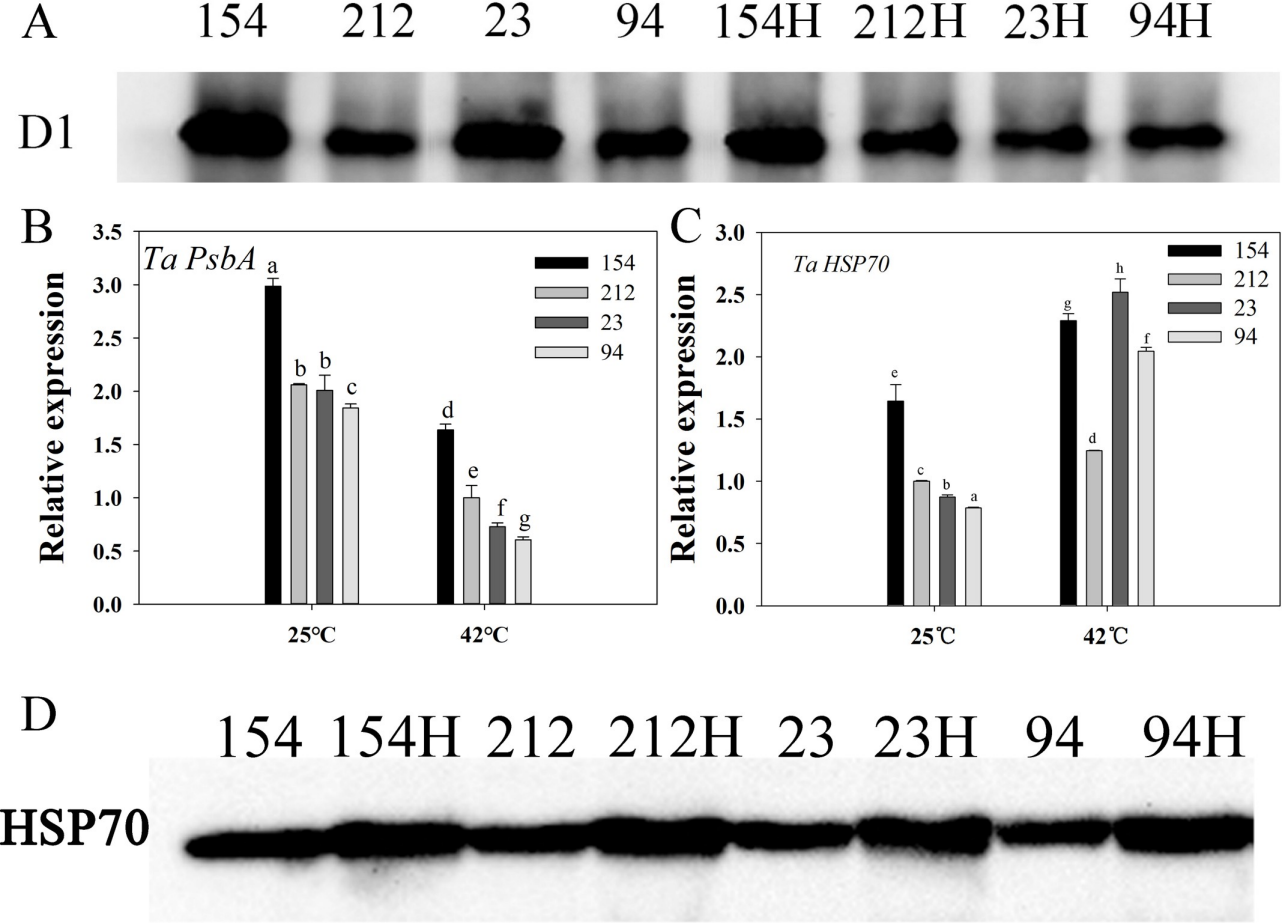
Accumulation and gene expression of D1 protein and heat shock protein 70 (HSP70) in NILs under high-temperature stress. (A) Western blot results of D1 protein, (B) gene expression of D1 (*PsbA*), (C) *HSP70* gene expression, (D) Western blot results of HSP70. Lines 154 and 23 possess high photosynthetic rates; Lines 212 and 94 possess low photosynthetic rates. The values are the mean ± SE from three independent experiments. Different letters indicate significant differences at P = 0.05.

De novo synthesis of the D1 protein, which forms the reaction center of PSII, is extremely important for the repair of PSII [46]; thus, we examined the accumulation of D1 protein and the expression of D1-related genes such as *TaPsbA*. The results showed that the D1 protein levels of the lines with high photosynthetic rates (154 and 23) were significantly higher than those of the lines with low photosynthetic rates (212 and 94) under normal conditions (Fig 5A). However, after heat treatment, the D1 protein levels declined in all NILs, but the high photosynthetic rate lines still maintained higher levels of D1 protein than the low photosynthetic rate lines (Fig 5A). Similar changes were also found in the expression of *TaPsbA* (Fig 5B).

We assessed the buildup of HSP70, which plays a key role in regenerating denatured proteins, maintaining cell homeostasis and shielding organisms from degradation in stressful environments [47]. After 4 h of high-temperature treatment, the expression of *HSP70* was significantly increased in all wheat lines. The HSP70 content of the high-Pn lines was still higher than that of the low-Pn lines; thus, the transcription level of the *HSP70* gene was consistent with the protein content results (Fig 5C and 5D).

These results indicate that high photosynthetic rate lines could maintain higher Pn and Calvin cycle key enzymatic activity, which may be due to the higher levels of D1 and HSP70 proteins contributing to stronger thermotolerance compared to lines with low photosynthetic rates.

### Antioxidative defense of NILs under high-temperature stress

**Fig 6:**
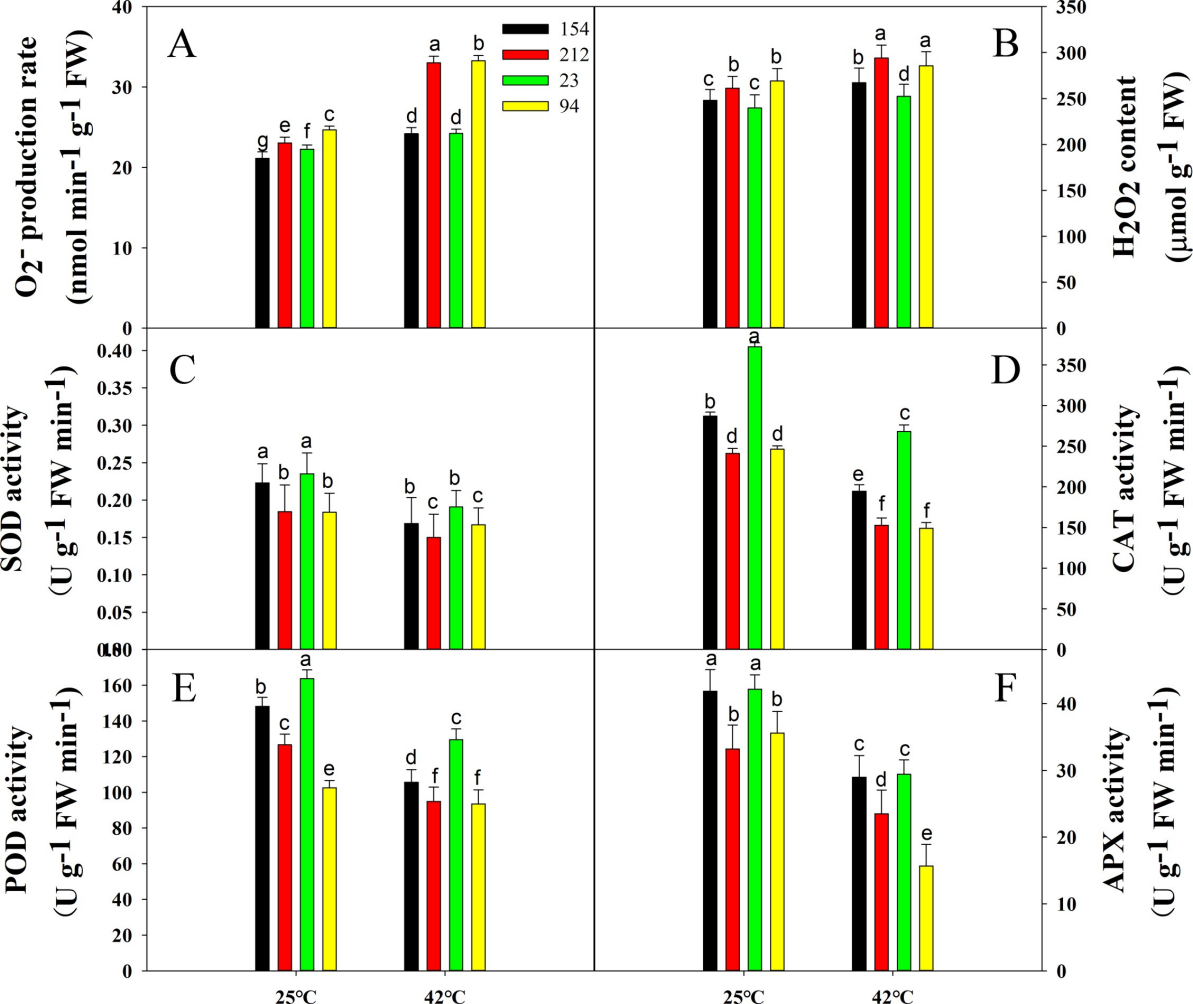
Effects of high temperature (treatment at 42°C for 4 h) on the contents of (A) O_2_·^−^ and (B) H_2_O_2_ and on the activities of antioxidant enzymes (C) SOD, (D) CAT, (E) POD, and (F) APX in flag leaves of wheat. Lines 154 and 23 possess high photosynthetic rates; Lines 212 and 94 possess low photosynthetic rates. Each bar represents the mean ± SE from three independent experiments. Different letters indicate significant differences at P = 0.05.

Plants generate massive quantities of ROS after adversity tension, such as hydrogen peroxide (H_2_O_2_) and radical superoxide (O_2_·^−^), triggering oxidizing cell membrane damage and assaulting the photosynthesis unit. Thus, we studied the amounts of H_2_O_2_ and O_2_·^−^ in natural and hot conditions in wheat leaves and found that prior to heat stress, lines with high photosynthetic rates (154 and 23) had lower contents of H_2_O_2_ and O_2_·^−^ than lines with low photosynthetic rates (212 and 94) (Fig 6A and 6B). However, after 42°C heat treatment for 4 h, the amounts of H_2_O_2_ and O_2_·^−^ increased considerably in both NILs, but the increase in the low photosynthetic rate lines was much higher than that in the high photosynthetic rate line lines (Fig 6A and 6B).

Next, in all wheat plants, we assessed the production of antioxidant enzymes, including catalase (CAT) and superoxide dismutase (SOD), as well as peroxidase and ascorbate peroxidase (APX), and we examined the cause of H_2_O_2_ or O_2_·^−^ (Fig 6C–6F). The results showed that lines with a high photosynthetic rate had higher activity of antioxidant enzymes under control and high-temperature conditions (Fig 6C–6F), which could explain why the lines with a high photosynthetic rate maintained a lower level of ROS.

**Fig 7:**
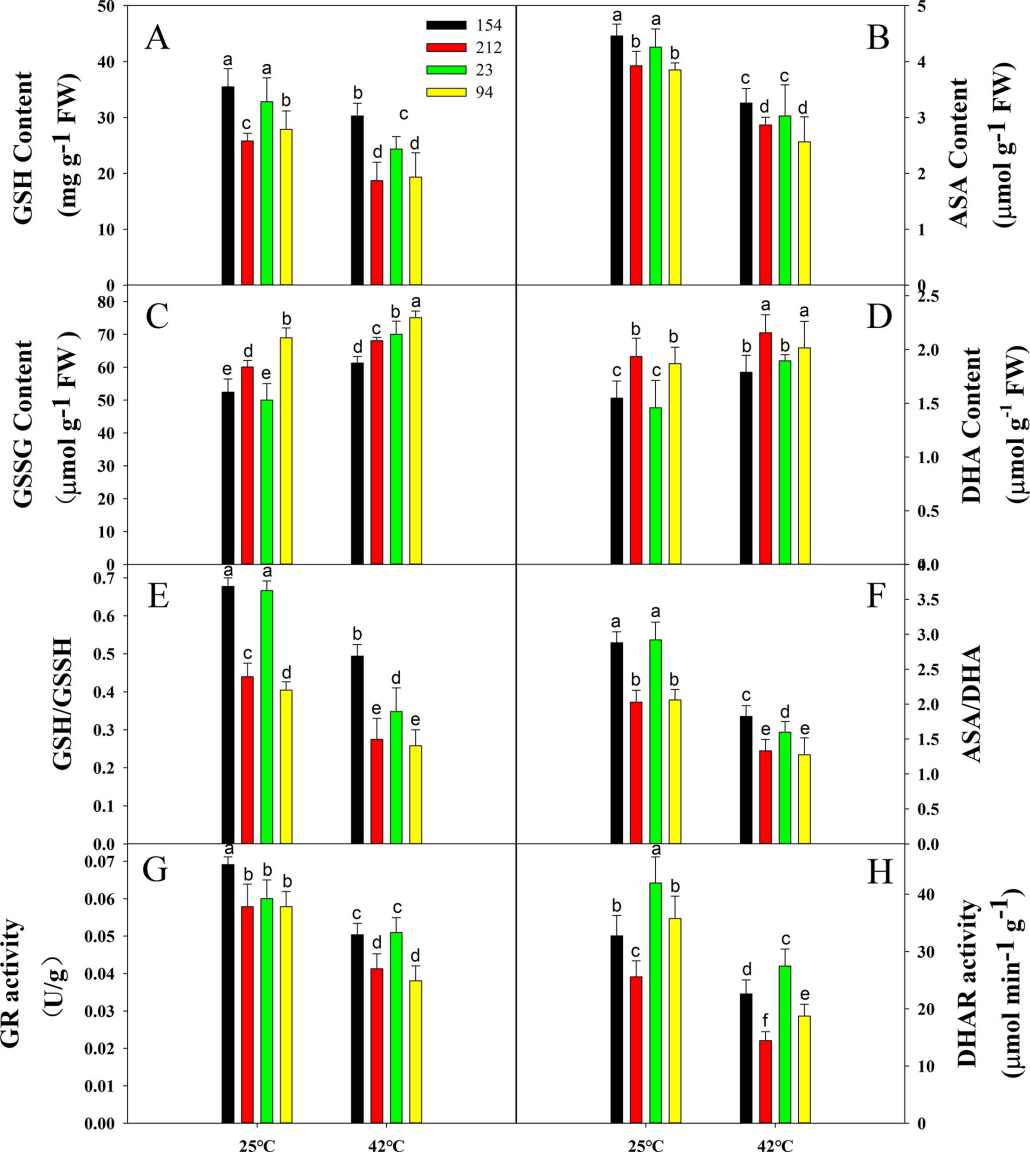
Effects of high temperature (treatment at 42°C for 4 h) on the contents of (A) GSH, (C) GSSG, (B) ASA, and (D) DHA; the ratio of (E) GSH/GSSG and (F) ASA/DHA; and the activities of (G) GR and (H) DHAR in wheat leaves. Lines 154 and 23 possess high photosynthetic rates; Lines 212 and 94 possess low photosynthetic rates. Each bar represents the mean ± SE of three independent experiments. Different letters indicate significant differences at P = 0.05.

Glutathione reductase (GR) and monodehydroascorbate reductase (DHAR) activities were also investigated, and the protein contents were heat-stressed, including glutathione (GSH), acid ascorbic acid (ASA), glutathiol (GSSG) and dehydroascorbic acid (DHA). The behaviors of GR and DHAR in both genotypes decreased considerably after 4 h of heat treatment but at a much greater rate in the lines with low photosynthetic rates (212 and 94). Moreover, although the contents of GSH and ASA, which are two major water-soluble antioxidants, decreased significantly in the NILs under heat stress, the high photosynthetic rate lines (154 and 23) still maintained a higher content than the low photosynthetic rate lines (212 and 94) (Fig 7). However, the content of GSSG and DHA was in contrast to the trend of GSH and ASA content (Fig 7A and 7B). Thus, the AsA/DHA and GSH/GSSG ratios in high photosynthetic rate lines (154 and 23) were higher than those of low photosynthetic rate lines (212 and 94) when exposed to 42°C for 4 h, indicating that the high photosynthetic rate lines (154 and 23) had a stronger ability to scavenge free radicals (Fig 7E and 7F).

## Discussion

Crop yields are extremely unpredictable and unsustainable because of the unpleasant climate [1] in dry and semiarid areas. Inadequate circumstances such as high temperatures in many parts of the world are an important agricultural issue. Wheat is a heat-sensitive crop; when under the influence of high temperatures, it undergoes many physiological changes that affect production [48]. The development of suitable NILs is important for studying plant genetic and physiological mechanisms [49].

In our study, between two pairs of wheat near isogenic lines with different photosynthetic rates (154 and 212 and 23 and 94), lines with high photosynthetic rates (154 and 23) could maintain higher Pn than lines with low photosynthetic rates (212 and 94) under normal conditions (Fig 1A), offering superior dry matter accumulation and grain yield in crop plants. Many experiments with antisense transgenic plants with decreased Calvin cycle enzymes have been carried out, and the results showed that decreases in the activities of these enzymes influenced CO_2_ fixation and inhibited photosynthesis [50–52]. Thus, the higher activities of key Calvin cycle enzymes, such as Rubisco, GAPDH, and sFBPase, in flag leaves contributed to maintaining the higher Pn in lines with a high photosynthetic rate under normal conditions (S1 Fig).

Two main photosynthesis reactions occur: the photoreaction, where light power is converted into ATP and NADPH and oxygen is released, and the dark reaction, which uses the light reaction to produce ATP and NADPH to immobilize CO_2_ into carbohydrates. Abiotic stress mainly affects crop yields by affecting light energy use and carbon dioxide fixation [53–55]. However, due to the different genotypes, it is difficult to further study the key processes and sites of photosynthesis that could allow lines with high photosynthetic rates to have strong thermotolerance.

In this research, after high-temperature therapy, the photosynthetic rate of both lines decreased considerably, whereas the Pn of lines with high photosynthetic rates was still higher than that of their peers (Fig 2A), indicating that lines with high photosynthetic rates could also have superior thermotolerance. Chlorophyll fluorescence is a specific method to detect plant health problems, photosynthesis and the impact on plants under abiotic stress [56–60]. Our results showed that the ΦPSII, Fv/Fm, and qP values significantly declined in all lines after high-temperature treatment, but the lines with high photosynthetic rates still maintained higher values of these chlorophyll fluorescence parameters than the lines with low photosynthetic rates (Fig 3A, 3B and 3D), suggesting that the degree of photoinhibition in the lines with high photosynthetic rates was slight and that the reduction in photosynthetic assimilation was correlated with a decrease in heat stress photochemical activity [61]. The NPQ results demonstrated that lines with a high photosynthetic rate can dissipate excess energy (Fig 3C), thereby protecting the photosystem to maintain a more stable PSII reaction center and maintain high photosynthetic capacity.

The Calvin cycle is centrally located in carbon metabolism, and manipulation of this route is likely to increase the crop yield and fixation of carbon [62]. Carbon fixation, however, is temperature-dependent and can be avoided by elevated temperatures. The decline in Rubisco behavior is related to a decrease in photosynthesis under moderate heat stress. Rubisco is thermally stable in higher plants, but under high-temperature stress, Rubisco activity is reduced. Yang, Liang [63] suggested that moderate heat stress inhibits Rubisco activase, and thus CO_2_ fixation is also inhibited. Interestingly, although the activities of key Calvin cycle enzymes (Rubisco, aldolase, GAPDH, and sFBPase) were significantly inhibited in all genotypes, the lines with high photosynthetic rates still maintained higher levels of enzymatic activity and related gene expression than the lines with low photosynthetic rates. These factors are closely related to the maintenance of higher Pn in lines with high photosynthetic rates during heat stress (Fig 4, S2 Fig).

In the photosynthetic apparatus of higher plants, the PSII reaction center is a key site for damage from high temperature, drought, and other stress injuries [64]. The degree of damage depends on its tolerance of adversity and its ability to repair damage [65]. PSII is a multisubunit protein complex, and D1 is the main target of adverse injury because it has a faster turnover rate than other proteins in the thylakoid membrane [66, 67]. Under normal conditions, the synthesis and degradation of D1 protein in higher plants are in dynamic equilibrium. Once exposed to bright light or glare and other adverse stress conditions, the degradation rate will exceed the D1 protein synthesis rate, resulting in destruction of the PSII reaction center [46, 68–70]. Therefore, the higher levels of D1 protein expression in lines with high photosynthetic rates could contribute to the protection of PSII, thereby maintaining strong thermotolerance after heat treatment (Fig 5A).

As a collaborator in protein evolution, HSP70 is a member of a high-molecular-weight community that protects it from aggregation/denaturation and contributes to the folding of new proteins [71–73]. HSP70 protects enzymes involved in the starch biosynthetic pathway under heat stress [74]. A relationship between heat stress and oxidative stress has been established, both of which lead to HSP70 expression [75]. Our findings show that in the lines with high photosynthesis, HSP70 expression increased more rapidly and was higher than that in the low photosynthetic rate lines after high-temperature stress (Fig 5C and 5D), which might be one of the reasons why the high photosynthesis lines maintained higher heat tolerance than the low photosynthesis lines.

While scavenging ROS, the antioxidant protection mechanism plays a significant role, protecting the photosynthetic apparatus against photoinhibition. The key source of ROS is a photosynthetic apparatus in the thylakoid membrane in photosynthetic cells, where O_2_·^−^, H_2_O_2_ and •OH are obtained through the transport of photosynthetic electrons [76–78]. Due to heat stress, ROS induce oxidation and exert major effects on the plant heat resistance of cellular biomolecules (which result in the oxidant degradation of proteins, membranes and nucleic acids) [21]. It is important for plant growth to establish a balance between the transportation of electrons in photosynthetic systems and the Calvin cycle [79]. The production of ROS takes place as chlorophyll consumes more light energy than can be used. CAT breaks down H_2_O_2_ into water and oxygen immediately. Increased activity is associated with increased tolerance to stress [80]. As with CAT, POD plays a vital role in the control of H_2_O_2_ levels via its conversion to H_2_O and its regeneration. POD also plays a major role in controlling H_2_O_2_ levels. The first line of protection against oxidative stress is generally considered SOD [81]. In this study, the decrease in antioxidant enzyme activity after high-temperature treatment may be due to heat stress, but the high photosynthetic rate lines still maintained higher activity levels of antioxidant enzymes (Fig 6) that could scavenge more ROS and reduce ROS accumulation, thereby protecting the photosynthetic machinery and maintaining a higher Pn under heat stress.

Another notable ROS mechanism is the ascorbate-glutathione cycle in chloroplasts. [77, 78]. Not only can the AsA-GSH cycle mitigate potential harm resulting from H_2_O_2_, but it can also help in the case of excess assimilation of electrons to maintain a certain degree of photosynthetic electron flow and reduce the potential harm to the optical system. AsA removes the electron acceptor of H_2_O_2_, and GSH acts as a common substrate for POD or as an active oxygen substrate against reactive oxygen species [82], in addition to promoting the ascorbic acid cycle. Moreover, AsA and GSH engage as enzyme substrates in metabolizing H_2_O_2_, directly quenching virtually all types of ROS and regulating photosynthesis redox status via an AsA-GSH cycle. Therefore, increased GR and DHAR operation and AsA and GSH content will help the high photosynthetic rate lines maintain a stronger ability to scavenge free radicals (Fig 7), thereby contributing to superior thermotolerance compared to low photosynthetic rate lines under high-temperature stress.

**Fig 8:**
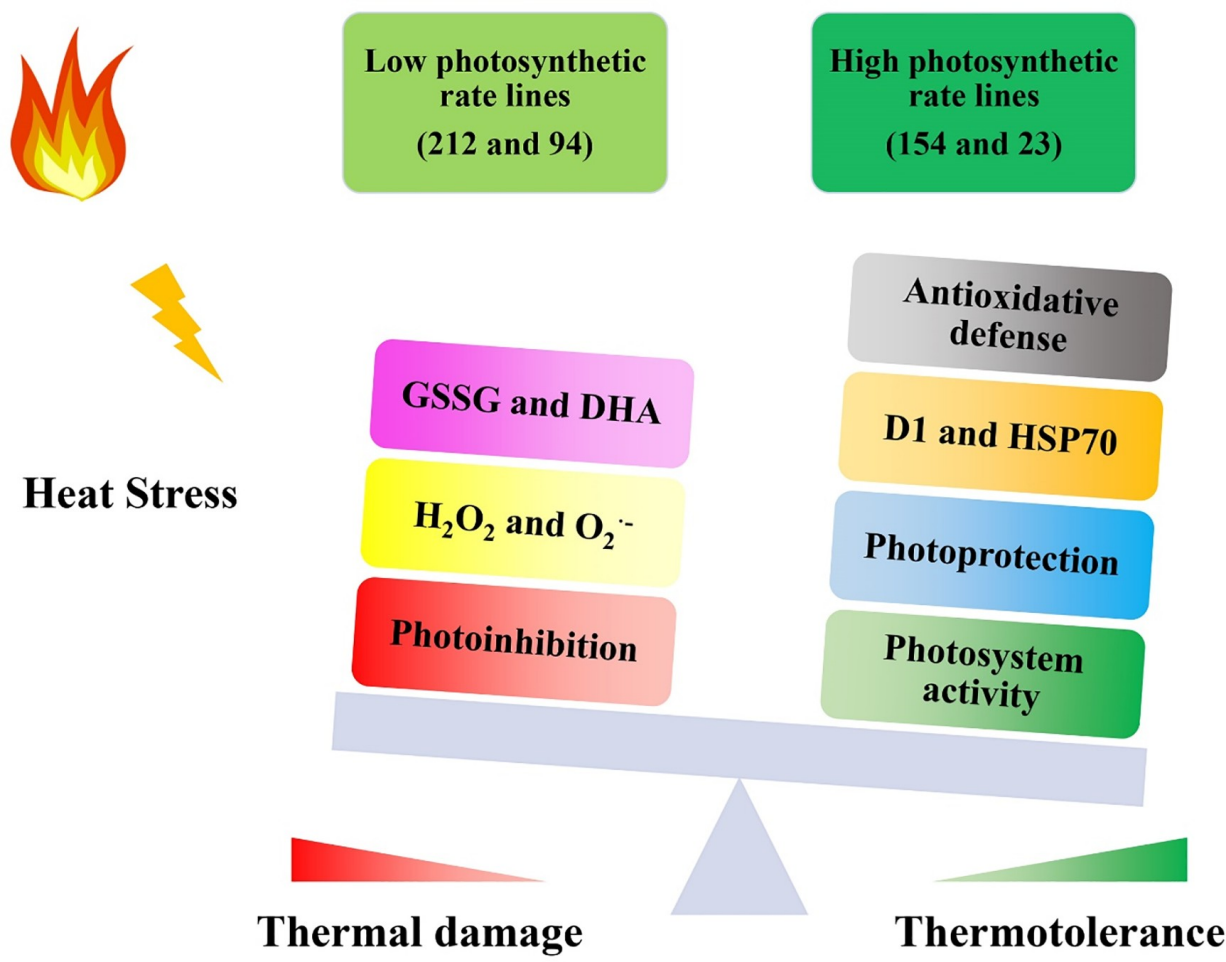
Model for mechanisms of heat stress tolerance in wheat NIL plants. Lines 154 and 23 possess high photosynthetic rates; Lines 212 and 94 possess low photosynthetic rates.

Based on the above results, a putative model is proposed in Fig 8. When wheat plants are shifted to a high temperature from the ideal temperature, thermal stress will increase excess aerosol energy and contribute to ROS output, photoinhibition and GSSG and DHA material. However, due to their increased activity, the antioxidant enzymes in highly photosynthetic lines can scavenge more ROS and reduce ROS accumulation to mitigate photoinhibition, thereby maintaining the stability of PSII and the integrity of the photosynthetic apparatus. Furthermore, the higher levels of D1 and HSP70 proteins can help high photosynthetic rate lines accelerate the repair of PSII and protect key Calvin cycle enzymes to maintain a higher Pn and ensure the stability of the Calvin cycle, thereby contributing to maintaining superior thermotolerance compared to low photosynthetic rate lines under high-temperature stress.

## Supporting information

S1 Table Sequences of primers (5’-3’) used for qRT-PCR analysis in wheat leaves with and without 42°C (4 h) heat stress. (DOCX)

S1 Fig Changes in the activity of (A) Rubisco, (B) GAPDH, (C) sFBPase, and (D) aldolase of flag leaves in the two pairs of NILs determined under normal conditions. Lines 154 and 23 possess high photosynthetic rates; Lines 212 and 94 possess low photosynthetic rates. The values are the mean ± SE from three independent experiments.
(TIFF)

S2 Fig Effects of high temperature (treatment at 42°C for 4 h) on the activities of key enzymes in the Calvin cycle of wheat measured by the gene expression of (A) Rubisco, (B) GAPDH, (C) sFBPase, and (D) aldolase. Lines 154 and 23 possess high photosynthetic rates; Lines 212 and 94 possess low photosynthetic rates. Each bar represents the mean ± SE from three independent experiments. Different letters indicate significant differences at P = 0.05.
(TIF)

S3 Fig (TIFF)

S4 Fig (TIFF)

S1 Data (XLSX)

S1 Raw image (PDF)
