# Supplementary material for: Comparison of photosynthetic activity and heat tolerance between near isogenic lines of wheat with different photosynthetic rates
Source: PLoS One. 2021 Dec 13;16(12):e0255896. doi: 10.1371/journal.pone.0255896 (PMC8668138; doi:10.1371/journal.pone.0255896)
Supplement: S1 Table — (DOCX) [file pone.0255896.s001.docx]

**Table S1** Sequences of primers (5’-3’) used for qRT-PCR analysis in wheat leaves with and without 42℃ (4 h) heat stress.

| Abbreviation | Primer sequence (5′-3′) |
| --- | --- |
| *Ta ACTIN-*F | CAAGGCGGAGTACGATGAGT |
| *Ta ACTIN-*R | AGTCCCCTTTGTAAGTCCCCT |
| *Ta PsbA-*F | AGTACAAGCCTGTGGGGTCGCT |
| *Ta PsbA-*R | AGGGGCAGCGATGAAGGCGATA |
| *Ta GAPDH-*F | TACACCCACGCCGACACCATCA |
| *Ta GAPDH-*R | TAGCCTCTGGTCGCCGGTGTAT |
| *Ta sFBPase -*F | TACATCGTGGTGTTCGACCCGC |
| *Ta sFBPase -*R | CGTCACTTCGTCAAGGGTTGCGT |
| *Ta Aldolase -*F | AGGCGTGTCAAAGAAGAGGGTAGCA |
| *Ta Aldolase -*R | TGAGGAGAGTAGCAGACGCCATTGT |
| *Ta Rubisco large subunit -*F | GGCTGCAGTAGCTGCCGAATCT |
| *Ta Rubisco large subunit -*R | TCCCCAGCAACAGGCTCGATGT |
| *Ta Rubisco small subunit-*F | AGCCTCAGCAGCGTCAGCAAT |
| *Ta Rubisco small subunit-*R | CGTGGATAGGGGTGGCAGGTAAGA |
| *Ta HSP70-*F | TGCAGATGCGGGTTGCTCGT |
| *Ta HSP70-*R | AGTTGGTGGTGCCCAGGTCGAT |
