# Supplementary figures and images for: Comparison of photosynthetic activity and heat tolerance between near isogenic lines of wheat with different photosynthetic rates
Source: PLoS One. 2021 Dec 13;16(12):e0255896. doi: 10.1371/journal.pone.0255896 (PMC8668138; doi:10.1371/journal.pone.0255896)

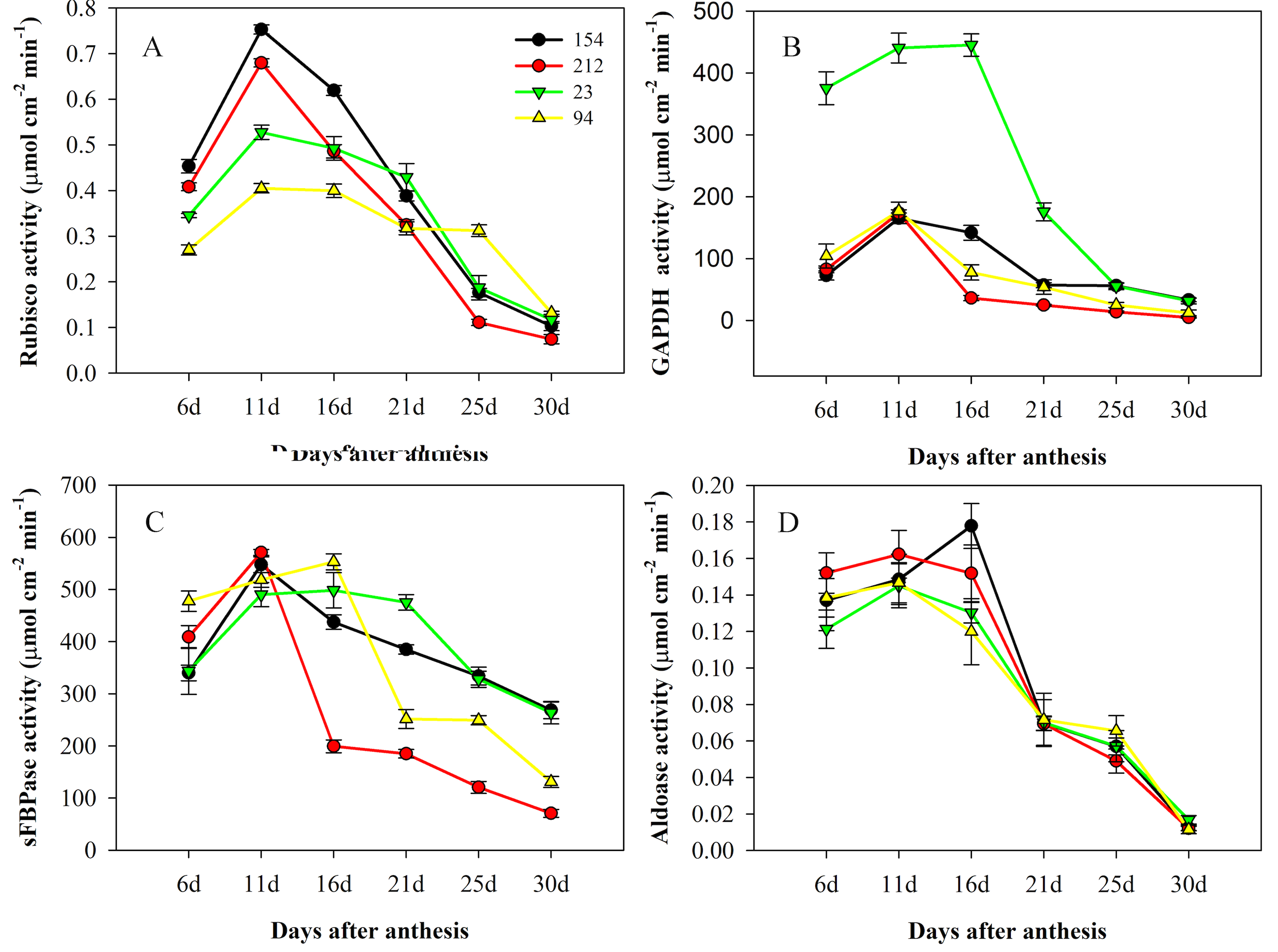

Supplement: S1 Fig — Changes in the activity of (A) Rubisco, (B) GAPDH, (C) sFBPase, and (D) aldolase of flag leaves in the two pairs of NILs determined under normal conditions. Lines 154 and 23 possess high photosynthetic rates; Lines 212 and 94 possess low photosynthetic rates. The values are the mean ± SE from three independent experiments. (TIFF) [file pone.0255896.s002.tiff]

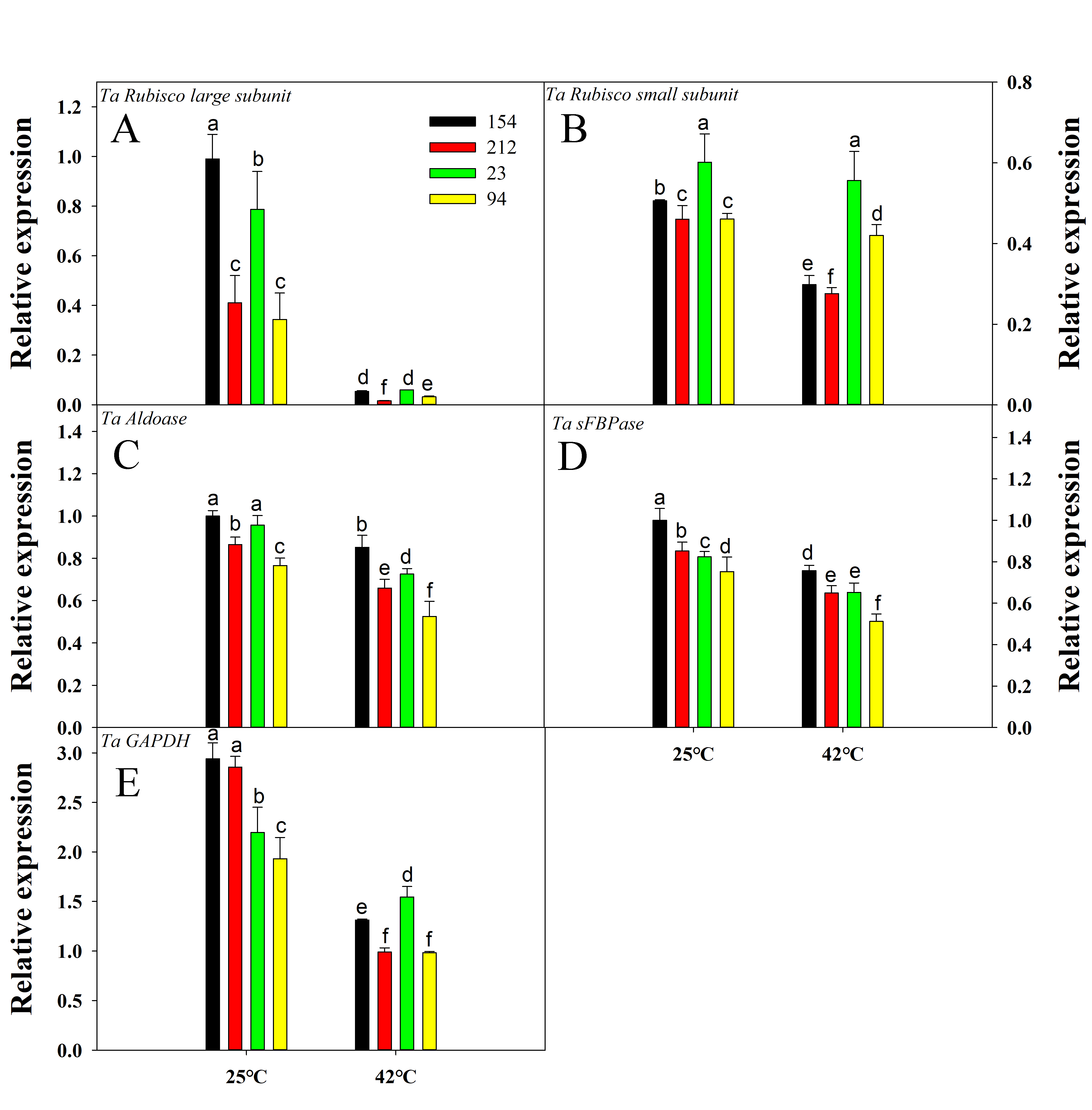

Supplement: S2 Fig — Effects of high temperature (treatment at 42°C for 4 h) on the activities of key enzymes in the Calvin cycle of wheat measured by the gene expression of (A) Rubisco, (B) GAPDH, (C) sFBPase, and (D) aldolase. Lines 154 and 23 possess high photosynthetic rates; Lines 212 and 94 possess low photosynthetic rates. Each bar represents the mean ± SE from three independent experiments. Different letters indicate significant differences at P = 0.05. (TIF) [file pone.0255896.s003.tif]

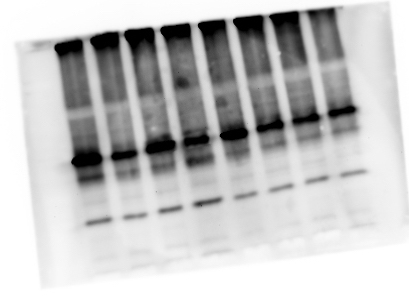

Supplement: S3 Fig — (TIFF) [file pone.0255896.s004.tiff]

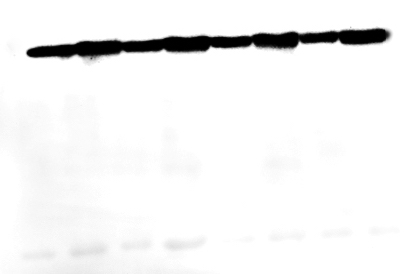

Supplement: S4 Fig — (TIFF) [file pone.0255896.s005.tiff]

**A**

154 212 23 94 154H 212H 23H 94H

**D1**

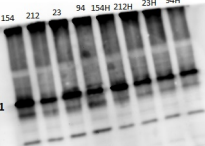

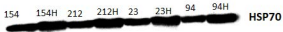

**B****Relative expression**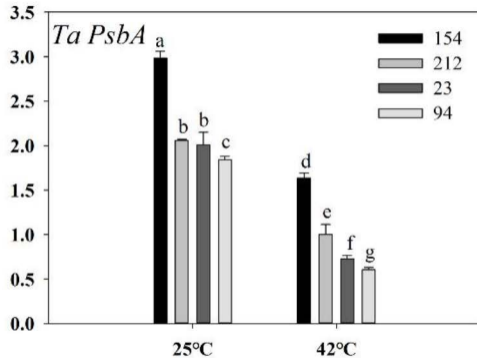**C****Relative expression**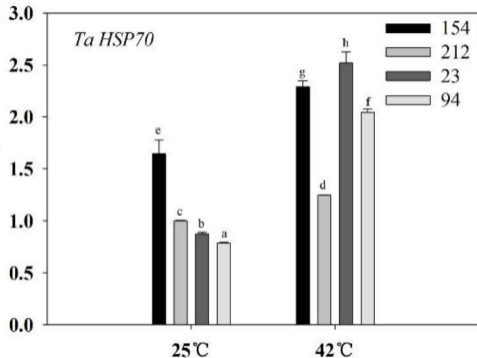

Supplement: S1 Raw image — (PDF) [file pone.0255896.s007.pdf]
